# How Long Halt Ye?

**Published:** 1921-01-08

**Authors:** Richard J. Coles

**Affiliations:** Secretary. King's College Hospital, Denmark Hill, S.E. 5


### THE EDITOR'S LETTER-BOX.

**Correspondence on all subjects is invited, but we cannot in any way be responsible for the opinions expressed by our correspondents, who must give their name and address as a guarantee of good faith, but not necessarily for publication. Correspondents are reminded that brevity of style and conciseness of statement greatly facilitate early insertion.**

### "How Long Halt Ye?"

*To the Editor of* THE HOSPITAL.

SIR,—I have read with interest Mr. Eason's letter to you which appeared in last week's issue of THE HOSPITAL. Like Mr. Glenton-Kerr, he has overlooked the fact that the so-called working-classes are not the only classes who should support the hospitals. There should be no exception so far as helping the hospitals is concerned. No one belittles what the Hospital Saturday Fund has done, and is doing, and, as stated in my previous letter, I will gladly do anything I can to assist the Fund's Council. Mr. Inman's letter in last week's issue is of great interest, and even he infers that we have to get beyond the so-called working-classes for support. We must appeal to the masses and not the classes. There must be no exceptions. It is not the time to be raising questions as to the desirability or otherwise of new versus old agencies for collecting. It is the time only for action and getting in the funds. To do this personal appeals must be made to the masses, and the workers in this great cause must be increased by thousands, aye, by hundreds of thousands, if we are to succeed.

I am glad to see that Mr. Inman points out the weakness of collecting-boxes in so far as workmen's collections are concerned. They are a failure because they *do not* record who contribute, and who do *not* do so. There is nothing like a systematic list of contributors who definitely agree to contribute a definite sum weekly, monthly, or otherwise. The best system of all is that where the employees, including clerks, shop assistants, accountants, members of other professions, and even domestics, request their employers to retain from their salary or wages at regular intervals the amount they have individually decided to give to the hospital.—Yours truly, RICHARD J. COLES, Secretary.

King's College Hospital, Denmark Hill, S.E. 5.
January 4, 1921.

